# Physiological Economy in Nutrition

**Published:** 1905-06-17

**Authors:** 


### Physiological Economy in Nutrition.

Diet and nutrition are subjects which have engaged the attention of medical men from the earliest times, but no valuable scientific work was published until about the middle of last century, when Carl Vöit of Munich placed on record the results of a number of experiments. According to Vöit, to maintain the body in equilibrium an adult of average weight doing moderate muscular work requires 118 grams of proteid, of which 105 grams should be absorbable, 56 grams of fat, 500 grams of carbo-hydrate. Subsequent investigators—Ranke, Moleschott and others—working on the same lines as Vöit, have arrived at results very much in accord with Vöit's, whose data have been accepted as a standard representing the needs of the body under normal conditions.

Dr. Chittenden of Yale University, in his book, "Physiological Economy in Nutrition," which has just been published by Messrs. Heinemann, challenges this standard, and states that as far as nitrogenous equilibrium is concerned it is too high.

We have carried out experiments to prove that, with reference to the minimal proteid requirement of the healthy man, a physiological economy in nutrition is possible. In planning these it was arranged that they should continue, not for a few days or weeks, but through months and years; also, as far as possible, monotony of diet was avoided, so that due recognition might be given to the psychical influences which, as Pawlow has shown, certainly do affect secretion. To minimise the influence of personal idiosyncrasy, three distinct classes of individuals were chosen. Firstly, a group of five men of varying ages, professors and instructors at the university, selected as representative of the mental rather than the physical worker. Secondly, thirteen volunteers from the Hospital Corps of the United States Army. These represent the moderate worker. Thirdly, a group of eight young university students, all trained athletes.

From careful and continuous observation on the different members of these groups Dr. Chittenden has arrived at some valuable conclusions. From his experiments on the first class of subjects he concludes that a much smaller amount of proteid or albuminous food than has hitherto been considered essential suffices to maintain the body and the nitrogen equilibrium without any loss of mental or physical strength. The average daily metabolism was 5·4–8·99 grams of nitrogen over a period of from six to 18 months.

Experiments with the 13 volunteers showed that the body-weight, nitrogen equilibrium, physical strength and vigour remain unimpaired under a daily diet involving the metabolism of only seven to eight grams nitrogen per diem. The men themselves said that they could do more work than formerly without feeling fatigue.

The athletes forming the third class believed, at the beginning of the experiments, that to maintain their strength and bodily vigour it was necessary to take large quantities of proteid food; they were allowed and encouraged to gradually diminish their diet at will. At the end of six months the table of results shows the amount of nitrogen excreted daily was 8.8 grams (55 grams proteid); the men were healthy and had gained in muscular power. The metabolised nitrogen per kilo of body-weight was fully as low as was obtained with members of the second class of subjects. These results are very important, and especially so because of the practical and thorough way in which they have been obtained. They point to the conclusion that the demand of the human body for proteid food does not exceed 50 per cent. of the usually accepted standard (118 grams). In connection with such a seemingly small albuminous or proteid diet, at least two questions will occur to many. Is a man working to the best advantage when he adheres to a formal standard of diet? Routine work may be satisfactorily accomplished, but is the desire for initiative, or the creative power of the brain so great, when it is attempted to maintain life on a minimum physiological diet? The second question relates to the disease resisting-power of the individual. Dr. Chittenden does not write definitely on this point, but he states that all the members of the three classes enjoyed good health and a remarkable freedom from illness while under observation. It is, indeed, a well-recognised fact that over-indulgence in proteid food is detrimental to health, but he would be a hardy man who would attempt an exact definition of the limit beyond which over-indulgence begins. Valuable and instructive as experiments are, the individual standard must depend largely on the person himself and his environment, because there are other issues to be considered apart from the physiological one.

